# Mosquito Passage Dramatically Changes *var* Gene Expression in Controlled Human *Plasmodium falciparum* Infections

**DOI:** 10.1371/journal.ppat.1005538

**Published:** 2016-04-12

**Authors:** Anna Bachmann, Michaela Petter, Ralf Krumkamp, Meral Esen, Jana Held, Judith A. M. Scholz, Tao Li, B. Kim Lee Sim, Stephen L. Hoffman, Peter G. Kremsner, Benjamin Mordmüller, Michael F. Duffy, Egbert Tannich

**Affiliations:** 1 Bernhard Nocht Institute for Tropical Medicine, Department of Molecular Parasitology, Hamburg, Germany; 2 Peter Doherty Institute, University of Melbourne, Department of Medicine, Melbourne, Victoria, Australia; 3 Bernhard Nocht Institute for Tropical Medicine and German Center for Infection Research, Infectious Disease Epidemiology Group, Hamburg, Germany; 4 Institute of Tropical Medicine and German Center for Infection Research, partner site Tübingen, Universitätsklinikum Tübingen, Tübingen, Germany; 5 Sanaria Inc., Rockville, Maryland, United States of America; 6 Centre de Recherches Médicales de Lambaréné, Lambaréné, Gabon; Hebrew University, ISRAEL

## Abstract

Virulence of the most deadly malaria parasite *Plasmodium falciparum* is linked to the variant surface antigen *Pf*EMP1, which is encoded by about 60 *var* genes per parasite genome. Although the expression of particular variants has been associated with different clinical outcomes, little is known about *var* gene expression at the onset of infection. By analyzing controlled human malaria infections via quantitative real-time PCR, we show that parasite populations from 18 volunteers expressed virtually identical transcript patterns that were dominated by the subtelomeric *var* gene group B and, to a lesser extent, group A. Furthermore, major changes in composition and frequency of *var* gene transcripts were detected between the parental parasite culture that was used to infect mosquitoes and *Plasmodia* recovered from infected volunteers, suggesting that *P*. *falciparum* resets its *var* gene expression during mosquito passage and starts with the broad expression of a specific subset of *var* genes when entering the human blood phase.

## Author Summary

Parasites of the species *Plasmodium falciparum*, which are responsible for the most severe forms of malaria, escape from the human immune response by antigenic variation. A repertoire of 60 *var* genes codes for a broad range of different variant antigens presented on the surface of infected erythrocytes. These antigens mediate adhesion to blood vessels, thereby disturbing the blood microcirculation and causing life-threatening organ dysfunctions. To better understand antigenic variation *in vivo* we analyzed the *var* gene expression profiles in blood samples from 18 malaria naïve volunteers, who were experimentally infected with cryopreserved sporozoites isolated from *Anopheles* mosquitoes. Our in-depth analysis revealed a broad, but remarkably uniform expression pattern of a specific set of *var* gene variants. Moreover, the results clearly show that this *var* gene expression program is specifically activated after mosquito transmission of the parasite. These findings are of particular importance for our understanding of the strategy of malaria parasites to establish and maintain infections in the human host.

## Introduction

Malaria is one of the most frequently occurring parasitic diseases worldwide with an estimated 198 million clinical cases in 2013 and a death toll of more than 0.5 million [1]. The virulence of the most deadly species of human malaria parasites, *Plasmodium falciparum*, is directly linked to the variable surface protein *Pf*EMP1 (*P*. *falciparum* erythrocyte membrane protein 1) [2,3]. Members of the *Pf*EMP1 family enable the parasite to adhere to a large variety of surface receptors on microvasculature linings or in case of pregnancy to the maternal side of the placenta in order to avoid spleen passage and subsequent clearance (reviewed in [4]). Expression switching between different *Pf*EMP1 variants correlates with changes in the antigenic and adhesion phenotype of the parasite [5]. Each parasite possesses about 60 *var* genes coding for different *Pf*EMP1 variants, which are expressed in a mutually exclusive manner meaning that generally only a single *Pf*EMP1 variant is exposed on the surface of the infected erythrocyte at a time while all other gene copies are silenced [6]. Historically, the global *var gene* repertoire present in the parasite population was assumed to be highly diverse and the number of variants almost unlimited. But in the recent past, evidence is arising that every parasite genotype is organized similarly and exhibits roughly the same numbers of *var* gene variants of each subgroup (A, B, C and E) defined by *Pf*EMP1 protein domain composition as well as by chromosomal localization, direction of transcription and particular 5’-UTR sequences of their encoding *var* genes [7–9]. Moreover, the comparison of seven *P*. *falciparum* genomes revealed 23 *Pf*EMP1 domain cassettes (DCs), which seem to form conserved recombination and receptor-binding units [10]. Both observations point to a more conserved *var* gene repertoire than previously assumed.

The reference strain NF54 possesses 10 group A *var* gene copies including the interstrain conserved subfamilies *var1* and *var3*, which are all located near the end of chromosomes and have a transcriptional direction towards the telomeres. With exception of the short *var3 Pf*EMP1 variants, A-type *Pf*EMP1 proteins have an extended, multiple domain composition and a non CD36-binding head structure consisting of a DBLα1 and a CIDRα1/β/γ/δ domain. Their pattern of expression has been linked to severe disease outcome [11–20]. The 37 member group B *var* genes are located closest to the telomere and transcribed towards the centromere and their expression has been associated with both severe and mild malaria [13,15,19–21]. Recently, expression of A- and B-type *var* genes encoding the interstrain conserved *Pf*EMP1 DCs 8 and 13, which bind the endothelial receptor EPCR, as well as the DC number 5, known to mediate PECAM1 binding, were linked to severe malaria in young children [18,22,23]. Interestingly, some genes are chimeras of group B and A or C *var* genes and are thought to represent intermediate groups between the major groupings [8]. In the NF54 genome, 4 and 9 members form these intermediate groups B/A and B/C, respectively, which all have a 5’-UTR characteristic for B-type *var* genes. In contrast to B/A genes, which are very similar in location and transcriptional orientation to group B genes, the chromosomal characteristics of group B/C genes are in common with group C genes. The 13 group C genes are located at chromosome internal clusters, transcribed towards the telomeres and possess a C-type 5’-UTR. C-type *Pf*EMP1 variants are known to be expressed in parasites causing asymptomatic infection and in long-term *in vitro* cultivated parasites [11,13,24–27]. Most B- and C-type *Pf*EMP1 proteins have a 4-domain extracellular structure including a CD36-binding head structure consisting of a DBLα0 and a CIDRα2–6 domain plus another DBL and CIDR domain [7,10].

Despite this large repertoire of variant surface proteins, the vast majority of *P*. *falciparum* infections do not lead to severe disease, suggesting that parasite sequestration is a well-adapted process and increased parasite transmission to mosquitoes outweighs losses due to host death. Hence, *Pf*EMP1 facilitates repeated and long-lasting infections of the human host even after repeated exposures. But, remarkably, particular *Pf*EMP1 subtypes appear to be specialized for infection of malaria naïve hosts where the interaction of *Pf*EMP1 with endothelial and circulating cells directly causes obstruction of blood circulation, which contributes along with immunopathology to organ failure. In malaria endemic areas, severe malaria mainly affects young children under the age of five lacking a sufficient immune response from previous *Plasmodium* infections [28]. Therefore, a better understanding of the *var* gene expression in malaria naïve individuals and of the mechanisms that control the expression of particular *Pf*EMP1 types is of particular importance. Three previous studies analyzed *var* gene expression in three experimentally infected naive individuals [29–31]. Peters *et al*. found a single dominant B-type *var* transcript in two volunteers, whereas Lavstsen *et al*. and Wang *et al*. detected a more broad activation of most *var* genes at the early onset of the blood infection. Based on these results two different strategies used by the malaria parasite to initiate an infection in the human host are currently discussed in the scientific community. The first model suggests that the parasites may use an ordered hierarchical *var* gene expression program, meaning that most of the parasites express a single *var* type in the first generation after egress from the liver. In the following replication cycles the parasite switches to other *var* gene variants determined by the intrinsic rate for each gene to be turned on or off. This would provide an efficient mechanism to evade the host's immune system in concordance with protection of the remaining *Pf*EMP1 variants from unnecessary exposure to the immune system [29,32]. The second concept postulates the early exploration of the suitability of the available host sequestration receptors. Accordingly, the parasite population released from the liver expresses all *var* genes and later on selective forces favor the survival of parasites expressing certain *Pf*EMP1 variants with the best adhesion properties and for which the human host has no pre-existing variant-specific immunity [30].

To clarify these contradicting concepts the study presented here made use of a controlled human malaria infection (CHMI) trial in which volunteers were injected with aseptic, purified, cryopreserved *P*. *falciparum* NF54 sporozoites (PfSPZ Challenge) of a single production lot produced under cGMP [33] and *var* gene expression was analyzed in samples from 18 malaria naïve hosts at the early onset of blood infection. The data presented here reveal a strategy, which favors the expression of a broad subset of *var* gene variants from subtelomeric locations while repressing *var* variants that are conserved between strains or that are located at chromosome internal sites. Moreover, we clearly show for the first time that *ex vivo var* gene expression patterns differ significantly from the expression profile of the parasite culture that was used to produce the PfSPZ Challenge lot used for CHMI, indicating that the expression of subtelomeric *var* genes is specifically turned on *in vivo*. Therefore our data support aspects of both postulated models: the parasite population seems to activate a whole subset of *var* genes allowing for exploration of the situation in the host which is shaped by available host receptors (e.g. CSA in pregnant women) and pre-existing variant specific immunity, whereas a different subset of *var* genes remains infrequently expressed.

## Results

### Infection kinetics of the volunteers

Our study was carried out in the frame of a dose-finding CHMI trial, in which malaria-naïve volunteers were infected with increasing doses of PfSPZ Challenge [33]. All individuals underwent CHMI with PfSPZ from the same lot of PfSPZ Challenge either by intravenous (iv, n = 24) or intradermal (id, n = 6) injections of 50 to 3,200 sporozoites. Samples from a subset of the volunteers inoculated with 200 to 3,200 sporozoites by intravenous injection (n = 15) or 2,500 sporozoites by intradermal injection (n = 3) were assessed (Table 1). All volunteers were examined every 12 hours from day 5 after PfSPZ injection and were treated immediately once their thick blood smear became parasite positive by microscopy. On average, the subset of volunteers included for *var* transcript analysis became parasite positive by thick blood smear ~12 days after infection (mean = 12.0; standard deviation (SD): ±1.2 days post infection) with parasitemias ranging between 2.5 and 54 parasites per μl of blood (mean = 9.1; SD: ±10.1 parasites/μl) (Table 1). In concordance with previous CHMI studies liver stage development took about 6 days and parasite kinetics measured by qPCR in this study suggest that parasites of the third generation after liver release were generally detected at day 11 and 12 post infection [30,33,34]. Accordingly, samples from volunteers infected intravenously either with 800 or 3,200 PfSPZ contained third generation blood phase parasites, whereas volunteer 02.1, the only one infected with 200 PfSPZ, and volunteers infected intradermally with 2,500 *Pf*SPZ had fourth and fifth generation blood phase parasites (day 13–15) (Table 1). The blood from 18 infected volunteers was preserved for transcript profiling of the entire NF54 *var* gene repertoire at the day of patent infection defined as presence of ring stage parasites in the thick blood smear.

**Table 1:** Overview of volunteer characteristics infected with PfSPZ Challenge.

Volunteer ID	Gender	No. ofPfSPZ	Route of administration	Time*	Parasites/μl**
02.1	m	200	iv	13.94	54
08.1	m	800	iv	10.88	2.5
08.2	f	800	iv	11.29	6
08.3	m	800	iv	11.99	6
08.4	f	800	iv	12.26	8.5
08.5	m	800	iv	12.08	9
25.1	f	2,500	id	15.34	11
25.2	m	2,500	id	13.87	8
25.3	m	2,500	id	12.89	3.5
32.1	m	3,200	iv	10.50	6.5
32.2	m	3,200	iv	11.05	7.5
32.3	m	3,200	iv	11.52	5.5
32.4	f	3,200	iv	11.11	6.5
32.5	m	3,200	iv	10.99	7.5
32.6	m	3,200	iv	11.00	10
32.7	m	3,200	iv	10.45	7
32.8	m	3,200	iv	11.55	9.5
32.9	f	3,200	iv	12.48	9

* Days between volunteer infection and positive thick blood smear (day malaria)

** Parasite density at diagnosis determined by thick blood smear

f: female; id: intradermal; iv: intravenous; m: male

### *Var* transcript levels in NF54 sporozoite infected volunteers at the day of patent infection

**Fig 1:**
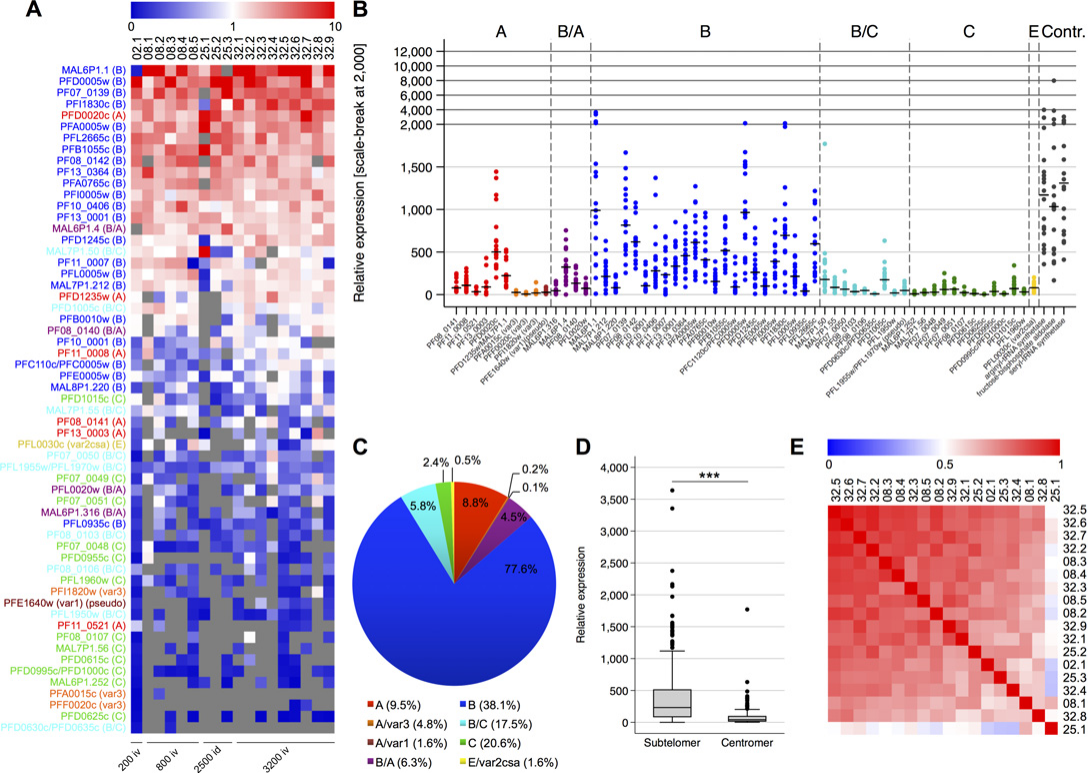
*Var* transcription profiles of parasites recovered from infected volunteers at the day of first microscopically detectable parasitemia. (A) Heat map showing the individual *var* gene expression for all volunteer samples taken when parasites were present in the thick blood smear ranked by mean expression. To correct for individual differences in the overall *var* expression levels, the expression for each *var* gene was normalized against the total *var* expression in each sample. The color scale indicates the relative expression levels with red representing values above the median, blue representing values below the median, and white representing median. Grey means not detected. The number of sporozoites (200, 800, 2500 and 3200) and mode of injection (iv = intravenous, id = intradermal) used for each volunteer are indicated below. (B) The distribution of the relative gene expression per *var* gene and control genes is shown in a dot plot for all volunteer samples at the day of patent infection defined as parasites present in the thick blood smear (n = 18). Each point represents a *var* gene expression value relative to the normalizing gene *sbp1* observed per volunteer sample and the median expression per *var* gene is marked. Housekeeping genes used as controls, *var* gene names and groups are indicated. (C) Proportion of *var* gene expression by group across all volunteers at the day of patent infection. For comparison, genomic proportion of each *var* gene group is indicated after the color code. (D) Comparison of the expression levels between subtelomeric and centromeric *var* gene variants. The box plot shows the distribution of transcript levels for each individual *var* gene relative to *sbp1* according to the chromosomal localization of the genes for all 18 volunteer samples at the day of patent infection. Gene expression varied significantly between both gene sets (Wilcoxon rank-sum test, p<0.0001) with median expression of 232 (IQR: 87–510) for telomeric *var* genes and 40 (IQR: 18–94) for centromeric *var* genes. (E) The heat map of pairwise Spearman’s rank correlation coefficients (R) between expression profiles illustrates the positive correlation between all 18 volunteer samples at the day of patent infection. Volunteer samples were ranked by the sum of their correlation coefficients. The color scale indicates the correlation coefficient in the range from 0 to 1. With exception of isolate 25.1 versus the isolates 02.1 (p = 0.0015), 25.3 (p = 0.0029) and 32.4 (p = 0.0030) all expressions correlated at a significance level below 0.001.

Analysis of *var* gene expression profiles in individual volunteers showed that transcripts of all *var* gene variants were detectable during the early blood stage NF54 infection. Interestingly, the individual gene expression profiles seemed to be very similar between the volunteer samples and the variability of the expression values for each *var* gene was mainly caused by differences in total *var* expression levels between the samples (Figs 1A and S1). In all samples a B-type variant was expressed at the highest level, mostly the variants MAL6P1.1/PF3D7_0632800 (IDs 08.1, 08.2, 08.4, 25.1, 32.2, 32.5 and 32.9) or PFD0005w/PF3D7_0400100 (IDs 02.1, 08.3, 25.2, 25.3, 32.1, 32.3 and 32.7) (Figs 1A and 1B and S2). Across all patients, the highest transcript levels were detected for MAL6P1.1/PF3D7_0632800 (median = 988.4; interquartile range (IQR): 479.2–1,535.9), followed by PFD0005w/PF3D7_0400100 (median = 964.5; IQR: 430.0–1,523.7), PF07_0139/PF3D7_0733000 (median = 814.9; IQR: 558.2–1,173.1), PFI1830c/PF3D7_0937800 (median = 695.8; IQR: 496.6–883.7) and PF08_0142/PF3D7_0800100 (median = 618.8; IQR: 464.0–667.7) (S2 Fig). All these genes are categorized as group B *var* genes, reflecting the dominant expression of group B *var* genes *in vivo* (Fig 1C). Conversely, transcript abundance of the *var* gene groups C and E showed some of the lowest expression levels detected. Within group A, the highest relative expression value was always detected for the EPCR-binding variant PFD0020c/PF3D7_0400400 (median = 500.5; IQR: 351.1–793.4), while the conserved group A *var1* pseudogene PFE1640w/PF3D7_0533100 and the three *var3* genes PFA0015c/PF3D7_0100300, PFI1820w/PF3D7_0937600 and PFF0020c/PF3D7_0600400 are among the 12 genes with lowest expression values (Figs 1A–1C and S2). A comparison of the expression levels between subtelomerically located *var* genes with those located on internal chromosome clusters revealed a significant difference between both gene subsets (Wilcoxon rank-sum test; p = <0.0001) (Fig 1D). Overall, transcript profiles showed high positive correlations between all patients, irrespective of infection route and dose (Figs 1E and S1). Furthermore, the observed expression profiles highly correlated with the expression profile obtained from a single volunteer in a previous study by Wang *et al*. [30] (Spearman’s Rank Correlation coefficient = 0.77, p < 0.001) (S3 Fig). In contrast, the observed *var* gene expression patterns showed no correlation with the data obtained from a previous study in which *var* gene activation from a null-*var* background induced by promoter-titration was monitored in an attempt to mimic the early onset of infection *in vitro* [35] (S3 Fig). In summary, the *var* gene expression pattern in NF54 parasites at the early onset of blood infection seems to be remarkably uniform at the level of both *var* gene groups and single *var* genes. In contrast to the high abundance of transcripts of a restricted subset of *var* genes which we previously observed in established clinical malaria cases [32], the expression profiles in parasites from all volunteers at this early stage of infection are rather broad without a single gene clearly dominating the infection (Fig 1A and 1B).

One interesting outlier from the general expression profile is the pattern detected in volunteer 02.1, in which the intermediate group B/A gene MAL6P1.4/PF3D7_0632500 was not expressed and transcript abundance of the neighboring B-type *var* gene MAL6P1.1/PF3D7_0632800 was significantly reduced in comparison to all other volunteer samples. Analysis of the genomic DNA by qPCR showed that this was due to a loss of the MAL6P1.4/PF3D7_0632500 gene from the entire parasite population and a partial loss of the MAL6P1.1/PF3D7_0632800 variant, which was only present in the genome of about 2.5% of the parasites in volunteer 02.1 (S4 Fig).

### *Var* transcript levels in PfSPZ Challenge-infected volunteers over time

**Fig 2:**
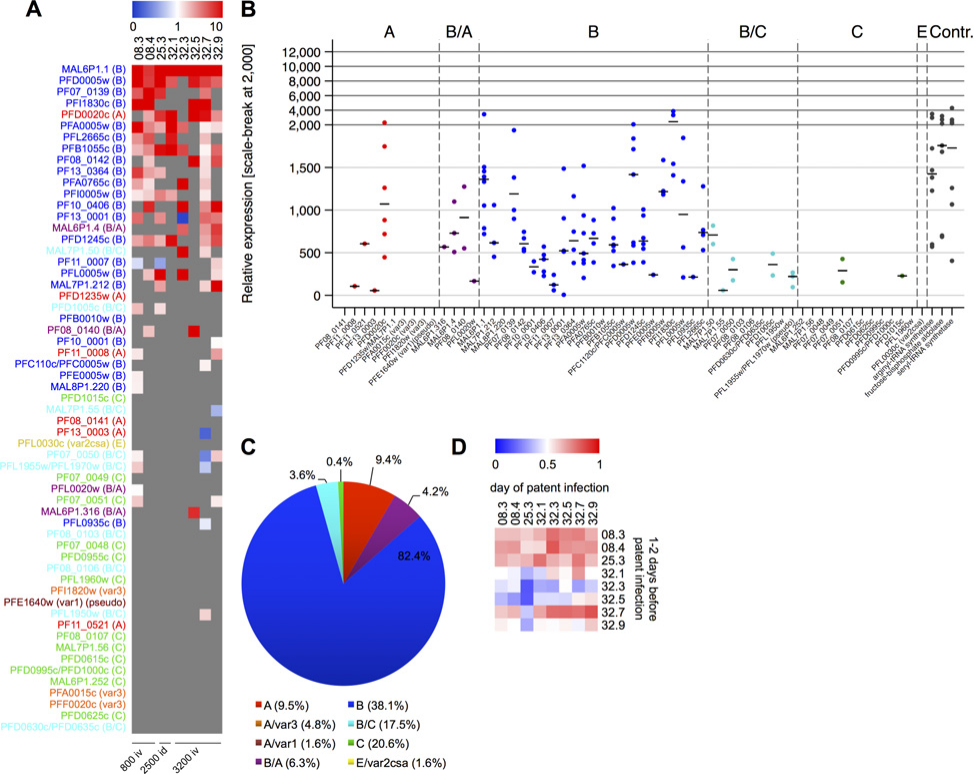
*Var* transcription profiles of parasites from infected volunteers 1–2 days before parasites were microscopically detectable. (A) Heat map showing the individual *var* gene expression profiles for samples obtained from volunteers one or two days before parasites were present in the thick blood smear. To correct for individual differences in the overall *var* expression levels, the expression for each *var* gene was normalized against the total *var* expression in each sample. Expression is ranked by mean expression obtained from “day of patent infection” samples (see Fig 1A). The color scale indicates the relative expression levels with red representing values above the median, blue representing values below the median, and white representing median. Grey means not detected. (B) Relative gene expression is shown in a dot plot for the volunteer samples one time point before thick blood smear positivity (n = 8). Gene IDs of *var* genes and controls are indicated on the x-axis, *var* gene groups are indicated above the graph. (C) The distribution of *var* transcripts according to *var* group affiliation in the volunteer samples 1–2 days before parasites were detected in the thick blood smear is displayed by summarization of the total *var* gene expression and calculation of the proportion for each *var* group. The genomic proportion of each *var* gene group is indicated after the color code. (D) The pairwise Spearman’s rank correlation heat map demonstrates a positive correlation between the expression profiles on the day of first microscopically detectable parasitemia and 1–2 days before in the same volunteer and between volunteer samples.

In addition to patient samples obtained at the day of first positive thick blood smear, samples were collected from 8 volunteers at time points with sub-microscopic parasitemia, prior to patent infection. *Var* transcript profiles were assessed on blood samples obtained either at day 9 (volunteers 32.1, 32.3, 32.5, 32.7) or day 11 post infection (volunteers 08.3, 08.4, 25.3, 32.9), thus, 1 or 2 days before the infected volunteer became microscopically positive (Fig 2A). Hence, the expression profiling only detected highly expressed variants because parasite load in these samples was extremely low (Fig 2A and 2B). qPCR confirmed high expression of B-type *var* genes and the A-type *var* gene PFD0020c/PF3D7_0400400 in these early samples (Fig 2A–2C). The expression profiles showed positive correlations between the two parasite generations *in vivo* (Fig 2D, Spearman’s Rank correlation) suggesting a stable *var* gene expression pattern during the first few blood phase parasite replication cycles at least in this group of malaria naïve individuals.

### Comparison of *var* transcript levels between pre-mosquito parasites and parasites recovered from infected volunteers at the day of patent infection

**Fig 3:**
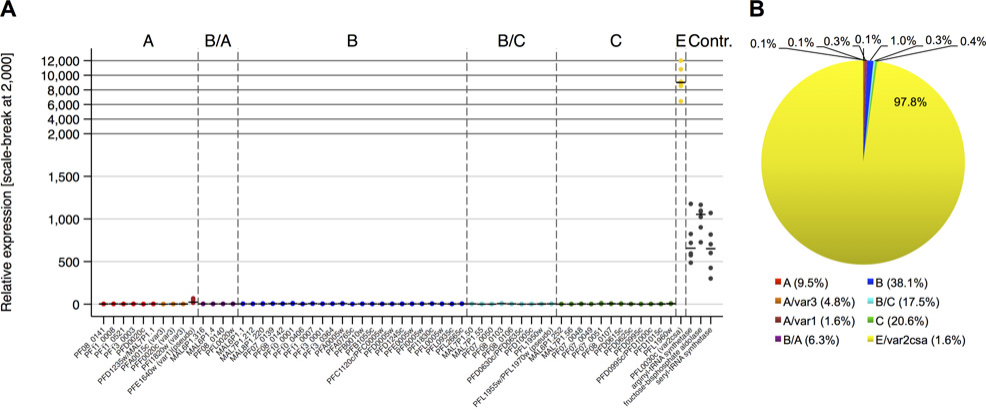
*Var* transcription profiles of parental parasite lines used for volunteer infection. (A) The gene expression of each *var* gene and control genes relative to *sbp1* expression is shown in dot plots for the pre-mosquito Master Cell Bank (MCB) parasite line. Each point represents values observed per test generation for the MCB samples taken from two independently thawed parasite stocks after 6, 8 and 21 parasite generations, respectively (n = 6). The median expression per *var* gene is shown, housekeeping genes used as controls, *var* gene names and groups are indicated. (B) The MCB cell line exclusively expresses the group E *var2csa* gene as shown by the proportion of *var* transcripts according to *var* group affiliation. The genomic proportion of each *var* gene group is indicated after the color code.

In order to investigate whether mosquito passage results in reprogramming of the *var* gene transcription pattern, we compared *ex vivo var* gene expression profiles in the volunteers with the *var* gene expression profiles in the parental parasite line used to produce the PfSPZ Challenge injected into the subjects. For this purpose, two vials of frozen NF54 parasites from the Sanaria Master Cell Bank RKV01-092505 (MCB) were independently thawed and cultured and *var* gene expression levels were analyzed after 6, 8 and 21 parasite replication cycles, respectively. The results indicate that the *in vitro*-adapted pre-mosquito NF54 line stably expressed exclusively the *var2csa* gene PFL0030c/PF3D7_1200600 (median = 9,012.7; IQR: 8,525.2–10,804.4) (Fig 3A and 3B).

**Fig 4:**
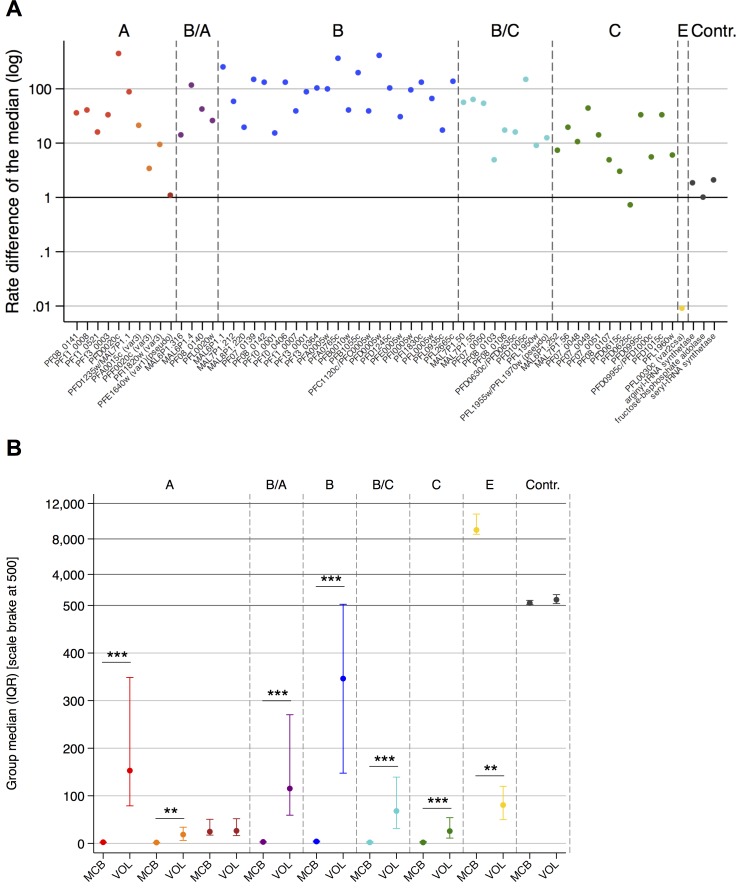
Modification of *var* transcription by mosquito transmission. (A) The rate difference of the median expression for each individual *var* variant and the housekeeping controls shows the overexpression of the entire *var* gene family *in vivo* with exception of the *var2csa* gene PFL0030c in comparison to the parental Master Cell Bank (MCB) parasite line. Each point reflects the median for the volunteer samples at the day of patent infection (n = 18) divided by the median observed for the parasite generations 6, 8 and 21 from two vials of the MCB cell line (n = 6). Housekeeping genes used as controls, *var* gene names and groups are indicated. (B) Differences in gene expression on group level between the pre-mosquito MCB parasite lines and parasites recovered from the infected volunteers (VOL) at the day of patent infection determined by thick blood smear are displayed as group median with interquartile range (IQR). Significant differences in distributions between MCB and VOL series were tested via a Wilcoxon rank-sum test using a Bonferroni corrected significance level. The graph contains a scale brake at the y-axis to account for the huge variability in the gene expressions. Group affiliations are indicated above the graph. Red (A), orange (A, subfamily *var3*), dark red (A, subfamily *var1*), purple (B/A), blue (B), turquoise (B/C), green (C) and yellow (E).

Given the abundant expression of *var2csa* in the parental NF54 parasites and the monoallelic expression of *var* genes [6,36] it was unsurprising that direct comparisons of transcript levels between the parental NF54 parasite line MCB and parasites recovered from the infected volunteers revealed increased expression *in vivo* of the entire gene family except *var2csa* (Fig 4A). The elevated expression of the *var* groups in the infected volunteer samples was significant; A (p <0.0001) including the *var3* subfamily (p = 0.0028), B/A (p <0.0001), B (p <0.0001), B/C (p <0.0001) and C (p <0.0001). The only exception was the decreased expression of the group E *var2csa* gene (p = 0.0045) and the *var1* subfamily pseudogene PFE1640w/PF3D7_0533100 was expressed at very low, unchanging levels in both NF54 and volunteers (Fig 4B). Thus, the *var* gene expression profiles of the *ex vivo* patient samples revealed substantial changes in comparison to the pre-mosquito NF54 parasites, which indicates epigenetic reprogramming of *var* genes during mosquito and/or liver passage.

## Discussion

Analyses of the role of *Pf*EMP1 in malaria pathogenesis and protection are hampered by the high sequence variability of the encoding *var* genes. Hence, quantitative expression data of the entire *var* gene repertoire from *P*. *falciparum* patient isolates are not well documented. The most common strategy for analyzing *var* gene expression in parasite strains with variable genomic background uses degenerate primer pairs targeting conserved sequence blocks for semi-quantitative RT-PCR or qPCR approaches. Using this strategy a higher frequency of A- and B-type *Pf*EMP1 transcripts was detected in malaria patients suffering from severe disease in comparison to mild or asymptomatic malaria [11–21]. In contrast to malaria patients who have already developed clinical symptoms of the disease and parasites ran through an unknown number of cycles, parasites from CHMI studies are only allowed to progress through a few, well-characterized replication cycles *in vivo* before the volunteers have to be treated. Accordingly, patients infected in CHMI studies have very low levels of parasitemia and do not develop complications (reviewed in [34]). Using samples from such a CHMI study we performed the first in depth quantitative analysis of *var* gene expression on 18 volunteer samples at the early onset of blood infection. All volunteers were infected with the NF54 strain and the same cryopreserved PfSPZ lot was used for intravenous or intradermal injection. In agreement with the expression of all or most of the NF54 *var* gene repertoire in parasites from recently infected volunteers express, there is also evidence that parasites tend to express many *var* genes rather than a single dominant variant in individuals with low levels of naturally acquired immunity [12,32,37]. Interestingly, genes possessing A- and B-type 5’-UTRs revealed a significant higher expression level in comparison to genes with C- or E-type 5’-UTR. Moreover, the expression level also seems to be influenced by the chromosomal position of the gene since the subtelomerically located A-, B/A- and B-type genes show higher expression levels than centromerically located genes of the B/C and C groups. This observation is in line with previously described differences in on- and off-rates for subtelomerically versus centromerically located *var* gene variants indicating that subtelomerically located variants have a much higher expression dynamic [25,36,38,39]. Lowest transcript abundances *in vivo* were detected for centromeric genes with C-type 5'-UTR. The only exceptions are the *var* gene variants of the *var1* and *var3* subfamilies, which show a very low expression level despite their A-type promoter sequence and their subtelomeric location. Together with the E-type *var2csa* (PFL0030c/PF3D7_1200600) gene all interstrain conserved *var* gene variants reveal only minor transcript abundances in all volunteers analyzed.

The first study on *var* gene expression in *ex vivo* samples of 3D7-infected volunteers applied a semi-quantitative RT-PCR strategy followed by sequencing of a large number of clones. On day 12.5 or 13.5 post infection most of the detected transcripts belonged to subtelomeric *var* genes of group A and B, consistent with the results presented here [29]. In contrast to the broad expression of most *var* genes detected in our study, a single B-type *Pf*EMP1 transcript, PF11_0007/PF3D7_1100100, was found most frequently and clearly dominated the expression profile in both volunteers analyzed by Peters and colleagues. One possible explanation for this divergent observation is the susceptibility of the semi-quantitative RT-PCR method to primer and cloning bias, which can result in overestimation of transcript frequencies. In another study from Lavstsen *et al*. parasites from six infected volunteers were analyzed by qPCR after approximately a month of *in vitro*-cultivation. In line with our data, all parasite lines established from first-generation parasites after liver release showed a similar *var* gene expression pattern and transcripts of all *var* genes could be detected [31]. In contrast to the data obtained in our study, 9 of the 10 lowest transcribed genes were classified as A or B/A *var* gene, which may be the result of the higher expression dynamic of these genes and/or the *in vitro*-cultivation of the parasites before analysis. More recently, NF54 parasites isolated from a Dutch volunteer in another CHMI study were analyzed using a more accurate qPCR approach [30]. Due to the very low parasitemia in CHMI studies the authors were able to analyze transcript abundances of the full NF54 *var* repertoire in a single blood sample only taken at day 11 after infection. Interestingly, apart from slight differences in frequencies, they observed exactly the same pattern of *var* gene transcription at the early onset of blood infection and, remarkably, the highest transcript levels were observed for the same group B *var* gene variant, MAL6P1.1/PF3D7_0632800 (synonym PFF1595c). Moreover, the three least polymorphic *var* gene subfamilies *var1*, *var2csa* and *var3* were also among the 10 lowest transcript levels in the Dutch volunteer. The positive correlation between both *in vivo* data sets was highly significant (S3 Fig). These highly reproducible results indicate that an intrinsic *var* gene expression program of the NF54 parasite exists which seems to drive higher levels of transcription of A- and B-type 5'-UTR controlled genes with the exception of the conserved *var1* and *var3* genes at the onset of blood infection. Later on, growth advantages of parasites best adapted to their host’s adhesion surfaces may select for parasites expressing particular *var* gene variants, subsequently resulting in the typical, dominant expression of one or a few *var* genes as we observed in natural infections. But whether this *var* gene expression pattern is a general strategy used by all parasite strains to establish human blood infections waits to be confirmed. Further work is also needed to address the question whether *var* gene expression patterns differ between parasites from experimentally infected malaria-naïve humans and those who already underwent natural or experimental *P*. *falciparum* infections and, accordingly, possess a pre-formed immune response against *Pf*EMP1 variants expressed in previous infection(s).

Another study reversibly silenced all endogenous *var* genes by promoter titration to mimic the moment when parasites enter the human blood phase [35]. In agreement with our data, two weeks after drug removal these “null-*var*” parasites with erased epigenetic memory from previous *in vitro* generations broadly activated all *var* genes. However, although A- and B-type variants are also activated, these parasites tend to express predominantly group C *var* genes, which becomes even more clearly after one and three month of cultivation. Accordingly, central *var* genes are the most highly activated genes *in vitro*, which is in clear contrast to the expression pattern we observe *in vivo* where subtelomerically located *var* genes dominate. Accordingly, Spearman’s rank correlation analysis showed no significant correlation between the data set from Fastman *et al*. and our *in vivo* data (S3 Fig). Maybe the observed pattern *in vivo* is the result of resetting plus *in vivo* selection while the resetting of the *var* gene repertoire by promoter titration is the result of the resetting alone. Additionally, because Fastman *et al*. found highly activated B and C-type *var* genes adjacent to rarely activated genes they concluded that the probability of *var* genes to be turned on or off are independent of their chromosomal location or promoter type *per se* and rather seems to be associated with intrinsic properties of each gene. Although we also found expression level differences between adjacent gene variants, e.g. PFI1820 and PFI1830, our *ex vivo* data show a clear association of the subtelomeric position as well as A- and, especially, B-type promoters with higher expression level.

The comparison between *var* gene expression in the pre-mosquito NF54 parasites and in the *ex vivo* patient samples revealed substantial changes indicating a kind of resetting of the *var* gene expression program during mosquito and/or liver passage of parasites. Pre-mosquito MCB parasites predominantly transcribed the *var2csa* gene PFL0030c/PF3D7_1200600 and this gene was also among the most abundant transcripts in Sanaria’s Working Cell Bank lot SAN02-073009, which was derived from the MCB and used to produce the PfSPZ Challenge lot for this particular volunteer infection (personal communication Matthias Frank), which is in clear contrast to the broad expression pattern of the subtelomeric *var* gene groups in the patient samples. Previous studies have indicated that mosquito passage leads to a shift in the transcribed *var* profile towards expression of subtelomeric genes [29] and promiscuous *var* transcription [30]. By using a parental parasite that appeared to exclusively express *var2csa* and robust and exhaustive methods to quantitate the entire *var* repertoire, we prove conclusively for the first time that the expressed *var* repertoire is dramatically reset following mosquito passage. Two mechanisms could explain these results: either the *var* epigenetic program is altered following passage through the mosquito leading to erasure of epigenetic memory and activation of subtelomeric *var* genes by first generation merozoites; or parental parasites expressing *var2csa* were selected against after exiting asexual replication *in vitro*. Although the latter explanation is possible it would mean that nearly all of the parental parasites would either fail to passage through the mosquito or to establish the infection in the human host. In fact, some parasites transcribing *var2csa* express no *Pf*EMP1 on the surface of the infected erythrocyte, because *var2csa* is often repressed at the level of translation [40]. Those parasites fail to adhere to endothelial receptors and would be rapidly cleared by the spleen. Resetting of the epigenetic *var* program following mosquito passage is supported by the observation that release of artificial repression of the entire *var* repertoire in asexual parasites leads to promiscuous *var* activation within the population [35], similar to the *var* profiles following mosquito passage that we and Wang *et al*. observed [30]. Furthermore, in NF54 sporozoites *var* gene expression is largely silenced, which is in agreement with a resetting of the *var* expression program during mosquito passage [41]. Our findings synthesize these various studies into a complete picture of mosquito passage causing epigenetic reprogramming to allow expression of any member of the *var* repertoire but with an apparent bias towards the subtelomeric *var* genes. Whether this bias was the consequence of phenotype selection in the first rounds of asexual replication remains unknown.

Interestingly, the modification of variable antigen expression by vector transmission has recently also been shown for the rodent malaria parasite *P*. *chabaudi* [42]. Spence and colleagues showed that the high virulence of serially blood-passaged parasites is attenuated by parasite transmission through mosquitos and this correlates with altered expression of the *cir* (*chabaudi interspersed repeats*) multi-gene family. Moreover, the activation of 114 *cir* variants (57%) after mosquito passage of *P*. *chabaudi* parasites closely reflects the broad activation pattern of all or most subtelomerically located *var* gene variants at the onset of blood infection in malaria naïve volunteers. Although the *pir* (*Plasmodium interspersed repeats*) multi-gene family is unique to *P*. *vivax*, *P*. *knowlesi* and the rodent clade of malaria parasites without any significant sequence similarity in the *P*. *falciparum* genome [43], the reprogramming of antigenic variant gene expression by vector transmission seems to be universal in the *Plasmodium* genus [29,42,44,45]. In summary, our data from this study clearly show differences in *var* gene expression before mosquito passage of the NF54 strain and early onset of blood infections, providing novel evidence for an epigenetic reprogramming or resetting of virulence gene expression during parasite transmission as proposed previously [29,32,42].

## Materials and Methods

### Ethics statement

The ethics committee of the University Clinic and the Medical Faculty of the University of Tübingen approved the study and the U.S. Food and Drug Administration Agency (FDA) provided regulatory oversight. Investigation of the *var* gene expression pattern in early blood stage infection was an exploratory objective of the trial. The study was conducted according to the principles of the Declaration of Helsinki in its 6th revision as well as International Conference on Harmonization–Good Clinical Practice (ICH-GCP) guidelines. The study registration code with ClinicalTrials.gov is NCT01624961. All volunteers, aged 18 to 45 years, provided written informed consent and understanding of the study and procedures was assessed with a quiz [33].

### CHMI trial and blood sampling

At the Institute of Tropical Medicine in Tübingen, Germany, healthy, malaria-naïve volunteers were infected with aseptic, purified, cryopreserved NF54 sporozoites (PfSPZ Challenge) from a single lot manufactured by Sanaria Inc., USA [33].

Blood samples for thick blood smears were taken daily from the onset of merozoite release at 5 days after sporozoite injection. Blood samples for *var* transcription profiling were taken at a 48 hours interval. Sampling continued until parasites were detected in thick blood smears when anti-malaria treatment was initiated. Then, erythrocytes from all infected volunteers were separated from leukocytes by Lymphoprep (Axis-Shield) gradient centrifugation followed by filtration through Plasmodipur filter (EuroProxima). In total, 4.5–9.0 ml of packed red blood cells were obtained from 18 volunteers at the day of first positive *P*. *falciparum* parasitemia by thick blood smear and from 8 volunteers at one time point before the thick blood smear was positive (1–2 days).

### Parasite cultivation

Two frozen vials (A and B) of NF54 parasites from Sanaria’s MCB lot RKV01-092505 were separately thawed and maintained in culture using a protocol adopted from Trager and Jensen [46]. A hematocrit of 5% was adjusted using human O+ red blood cells and the parasite culture medium was supplemented with 10% heat-inactivated human serum.

### DNA purification

For DNA purification, MCB parasites were cultivated for 6 parasite replication cycles to obtain a higher yield. Genomic DNA was prepared from ring stage parasites established from both cell stocks using the QIAamp DNA Blood Mini Kit (Qiagen).

### RNA purification and cDNA synthesis

For RNA purification ring stage parasites from MCB vials A and B were taken after 6, 8 and 21 parasite replication cycles after thawing, respectively. One cycle of invasion before harvesting, parasite growth was synchronized twice at an interval of 6 hours using sorbitol [47]. Cell pellets of MCB and of the leukocyte depleted patient blood samples were rapidly lysed in 10 volumes pre-warmed TRIzol (Invitrogen) and stored at -80°C. RNA purification was performed according to the manufacturers instruction of the PureLink RNA Kit (Life Technologies) including DNase treatment on the column. Absence of DNA was checked using 50 ng RNA as template for a qPCR run with the *sbp1* primer pair. If necessary, DNasing was repeated until the sample was free of any DNA contamination by qPCR. Afterwards, cDNA was synthetized with the SuperScript III Reverse Transcriptase (Invitrogen) primed with random hexamers (Invitrogen) at 50°C for 1 hour. As possible, a cDNA synthesis reaction without enzyme was performed and analyzed in parallel by qPCR.

### Quantitative real-time PCR

Quantitative amplification was conducted in the ABI PRISM 7900HT Sequence Detection System (Applied Biosystems) using the SDS software version 2.3 (Applied Biosystems). Template was mixed with the SYBR Green PCR Master Mix (Applied Biosystems) and 0.3 μM forward and reverse primer in a final volume of 10 μl. Reactions were incubated at 50°C for 2 min, at 95°C for 10 min, then subjected to 40 cycles of 95°C for 15 s and 60°C for 1 min and a subsequent melting step (60–95°C). The specificity of each primer pair was confirmed after each qPCR run by dissociation curve analysis.

Analysis was performed using *sbp1* (PFE0065w/PF3D7_0501300) to normalize and gDNA from MCB parasites to calibrate the individual *var* gene expression data. Relative quantification of the NF54 *var* repertoire by 2^-ΔΔCt^ analysis was performed using a previously described primer set [48] supplemented with new primer pairs for PF07_0049/PF3D7_0712000, PFL1950w/PF3D7_1240300, PFE0005w/PF3D7_0500100 and PF07_0051/PF3D7_0712600. Furthermore, primer pairs targeting the housekeeping genes *fructose-bisphosphate aldolase* (PF14_0425/PF3D7_1444800), *seryl-tRNA synthetase* (PF07_0073/PF3D7_0717700) and *arginyl-tRNA synthetase* (PFL0900c/PF3D7_1218600) were included (S1 Table). Relative expression data were corrected for amplification efficiency of each primer pair, which was determined by dilution of a single gDNA from 3D7 over 5 logs of concentration (S1 Table). Expression and correlation heat maps were generated using Multiple Experiment Viewer (MeV). The expression plots were programmed with Stata version 14. In all expression dot plots a scale break of the y-axis was introduced at 2,000 due to the huge difference of the relative expression values between gene groups.

### Statistical analysis

Spearman’s rank correlation was applied to assess whether gene expression patterns between parasites isolated from different infected volunteers or between different parasite generations from the same infected volunteer were comparable. Correlation coefficients (R-values) were displayed in a heat-map, where color codes define the correlation levels (Figs 1E and 2D). Furthermore, Spearman’s rank correlation was used to compare the gene expression patterns observed in this study with the results obtained by Wang *et al*. [30] and Fastman *et al*. [35] (S3C Fig).

To compare *var* gene expression levels between infected volunteers, and the pre-mosquito cell line MCB differences in the median gene expressions were calculated for each individual *var* gene variant. Therefore, the median ratio was calculated. A value of 1 indicates no difference, >1 higher gene expression in the infected volunteers and a value <1 higher gene expression in the MCB cell line (Fig 4A).

To describe the expression of genes with different chromosomal localizations or per *var* gene group observations within respective groups were pooled and the median expression along with interquartile rage (IQR) was calculated. Differences in gene expressions between the respective groups were tested via a Wilcoxon rank sum test. This analysis was applied to assess differences in gene expression levels of subtelomerically versus centromerically located *var* gene variants in the volunteer samples taken at the day of first microscopically detectable parasitemia (Fig 1D). Furthermore, expression level differences of *var* gene groups (i.e., A, A *var3* and *var1* subfamilies, B/A, B, B/C, C, E) and control genes between volunteer samples at the day of patent infection versus the pre-mosquito parasite line MCB were also tested via Wilcoxon rank sum test (Fig 4B). Bonferroni corrected significance level was used to account for multiple comparisons. Since comparison was done among 9 *var* gene groups, the p-value was corrected by the multiplication with 9.

All analyses were done using STATA 14 (College Station, TX: StataCorp LP) or GraphPad Prism 4.

## Supporting Information

S1 Table Primer sets used for qPCR analysis to amplify the entire NF54 *var* gene repertoire. (PDF)

S1 Fig Scatter plots of individual expression profiles against mean *var* gene expression from all *ex vivo* volunteer samples at the day of patent infection. Spearman’s rank correlation coefficient (R) and significances (p-values) are indicated above each graph.
(PDF)

S2 Fig Ranking of *var* genes according to expression level detected in samples from experimentally infected humans at the day of thick blood smear positivity. The median *var* transcript level relative to the *sbp1* transcript level with IQR is shown for all 18 volunteer samples. Expression values for genes of the different *var* groups are presented in red (A), orange (subfamily *var3*), dark red (subfamily *var1*), purple (B/A), blue (B), turquoise (B/C), green (C) and yellow (E).
(PDF)

S3 Fig Comparison of *ex vivo var* gene expression profiles in human volunteers to patterns observed in previous studies of a volunteer infection [30] and of epigenetically erased and reactivated *var* gene expression *in vitro* [35]. (A) Heat map showing the individual *var* gene expression profiles for all volunteer samples on the day of patent infection and for the volunteer sample obtained by Wang *et al*. [30]. Furthermore, *var* gene expression data is shown for two 3D7 cell lines (E1 and G6), in which a “null-*var*” phenotype was created *in vitro*, *var* gene expression was reactivated and analyzed two weeks, one and three months after drug removal [35]. *Var* genes are ranked by *var* gene group and mean expression. To correct for individual differences in the overall *var* expression levels and for the use of different normalizing genes in the three studies, the expression of each *var* gene was normalized against the total *var* expression in each sample. Samples were hierarchically clustered using average linkage clustering with Spearman’s Rank Correlation as distance metric. The color scale indicates the relative expression levels with red representing values above the median, blue representing values below the median, and white representing median. Grey means not detected. (B) Scatter plots of normalized *var* gene expression data obtained by Wang *et al*. [30] and Fastman *et al*. [35] against mean *var* gene expression from all *ex vivo* volunteer samples at the day of first microscopically detectable parasitemia. Spearman’s rank correlation coefficient (R) and significances (p-values) are indicated above each graph. (C) Heat map of pairwise Spearman’s rank correlation coefficients (R) of *var* gene expression profiles from Wang *et al*. and Fastman *et al*. [30,35] against the mean *ex vivo var* gene expression of all volunteers at the day of patent infection. The color scale indicates the correlation coefficient with red indicating positive correlation, blue indicating negative correlation and white indicating no correlation.
(PDF)

S4 Fig Parasites with deletion on chromosome 6 were recovered from volunteer 02.1. (A) *var* gene expression in parasites recovered from volunteer 02.1, who received 200 sporozoites intravenously 14 days before. Shown are transcript levels of all NF54 *var* genes and three housekeeping controls as indicated. For comparison, the median expression value calculated from all volunteer samples is displayed for each gene as a black line. Both genes with remarkably low expression values, MAL6P1.4/PF3D7_0632500 and MAL6P1.1/PF3D7_0632800, are highlighted in red boxes. (B) *var* qPCR analysis with genomic DNA extracted from two vials of Master Cell Bank parasites (MCB-A and -B), Working Cell Bank parasites (WCB) and parasites recovered from volunteers 02.1 (VOL 02.1) and 32.5 (VOL 32.5). Shown are Ct (cycle of treshold) values for each individual *var* gene primer pair. The primer pair for the variant MAL6P1.4 failed to detect an amplification product in the sample from volunteer 02.1 within 40 PCR cycles (Ct ≥ 40). An increased Ct value for the variant MAL6P1.1 of 5.3 PCR cycles reflects a reduction of 38.9 fold on genomic DNA level (2^ΔCt^).
(PDF)

## References

[1] World Health Organisation (2014) World Malaria Report 2014.

[2] Baruch DI, Pasloske BL, Singh HB, Bi X, Ma XC, et al (1995) Cloning the *P*. *falciparum* gene encoding PfEMP1, a malarial variant antigen and adherence receptor on the surface of parasitized human erythrocytes. Cell 82: 77–87. 7541722 10.1016/0092-8674(95)90054-3

[3] Su XZ, Heatwole VM, Wertheimer SP, Guinet F, Herrfeldt JA, et al (1995) The large diverse gene family *var* encodes proteins involved in cytoadherence and antigenic variation of *Plasmodium falciparum*-infected erythrocytes. Cell 82: 89–100. 7606788 10.1016/0092-8674(95)90055-1

[4] Rowe JA, Claessens A, Corrigan RA, Arman M (2009) Adhesion of *Plasmodium falciparum*-infected erythrocytes to human cells: molecular mechanisms and therapeutic implications. Expert Rev Mol Med 11: e16 10.1017/S1462399409001082 19467172 PMC2878476

[5] Smith JD, Chitnis CE, Craig AG, Roberts DJ, Hudson-Taylor DE, et al (1995) Switches in expression of *Plasmodium falciparum var* genes correlate with changes in antigenic and cytoadherent phenotypes of infected erythrocytes. Cell 82: 101–110. 7606775 10.1016/0092-8674(95)90056-x PMC3730239

[6] Voss TS, Healer J, Marty AJ, Duffy MF, Thompson JK, et al (2006) A *var* gene promoter controls allelic exclusion of virulence genes in *Plasmodium falciparum* malaria. Nature 439: 1004–1008. 16382237 10.1038/nature04407

[7] Kraemer SM, Smith JD (2003) Evidence for the importance of genetic structuring to the structural and functional specialization of the *Plasmodium falciparum var* gene family. Mol Microbiol 50: 1527–1538. 14651636 10.1046/j.1365-2958.2003.03814.x

[8] Lavstsen T, Salanti A, Jensen AT, Arnot DE, Theander TG (2003) Sub-grouping of *Plasmodium falciparum* 3D7 *var* genes based on sequence analysis of coding and non-coding regions. Malar J 2: 27 14565852 10.1186/1475-2875-2-27 PMC222925

[9] Kraemer SM, Kyes SA, Aggarwal G, Springer AL, Nelson SO, et al (2007) Patterns of gene recombination shape *var* gene repertoires in *Plasmodium falciparum*: comparisons of geographically diverse isolates. BMC Genomics 8: 45 17286864 10.1186/1471-2164-8-45 PMC1805758

[10] Rask TS, Hansen DA, Theander TG, Gorm Pedersen A, Lavstsen T (2010) *Plasmodium falciparum* erythrocyte membrane protein 1 diversity in seven genomes-divide and conquer. PLoS Comput Biol 6. 10.1371/journal.pcbi.1000933 PMC2940729 20862303

[11] Jensen AT, Magistrado P, Sharp S, Joergensen L, Lavstsen T, et al (2004) *Plasmodium falciparum* associated with severe childhood malaria preferentially expresses PfEMP1 encoded by group A *var* genes. J Exp Med 199: 1179–1190. 15123742 10.1084/jem.20040274 PMC2211911

[12] Bull PC, Berriman M, Kyes S, Quail MA, Hall N, et al (2005) *Plasmodium falciparum* variant surface antigen expression patterns during malaria. PLoS Pathog 1: e26 16304608 10.1371/journal.ppat.0010026 PMC1287908

[13] Kaestli M, Cockburn IA, Cortes A, Baea K, Rowe JA, et al (2006) Virulence of malaria is associated with differential expression of *Plasmodium falciparum va*r gene subgroups in a case-control study. J Infect Dis 193: 1567–1574. 16652286 10.1086/503776 PMC2877257

[14] Kyriacou HM, Stone GN, Challis RJ, Raza A, Lyke KE, et al (2006) Differential *var* gene transcription in *Plasmodium falciparum* isolates from patients with cerebral malaria compared to hyperparasitaemia. Mol Biochem Parasitol 150: 211–218. 16996149 10.1016/j.molbiopara.2006.08.005 PMC2176080

[15] Rottmann M, Lavstsen T, Mugasa JP, Kaestli M, Jensen AT, et al (2006) Differential expression of *var* gene groups is associated with morbidity caused by *Plasmodium falciparum* infection in Tanzanian children. Infect Immun 74: 3904–3911. 16790763 10.1128/IAI.02073-05 PMC1489729

[16] Warimwe GM, Keane TM, Fegan G, Musyoki JN, Newton CR, et al (2009) *Plasmodium falciparum var* gene expression is modified by host immunity. Proc Natl Acad Sci U S A 106: 21801–21806. 10.1073/pnas.0907590106 20018734 PMC2792160

[17] Lavstsen T, Turner L, Saguti F, Magistrado P, Rask TS, et al (2012) *Plasmodium falciparum* erythrocyte membrane protein 1 domain cassettes 8 and 13 are associated with severe malaria in children. Proc Natl Acad Sci U S A 109: E1791–1800. 10.1073/pnas.1120455109 22619319 PMC3387094

[18] Bertin GI, Lavstsen T, Guillonneau F, Doritchamou J, Wang CW, et al (2013) Expression of the domain cassette 8 *Plasmodium falciparum* erythrocyte membrane protein 1 is associated with cerebral malaria in Benin. PLoS One 8: e68368 10.1371/journal.pone.0068368 23922654 PMC3726661

[19] Almelli T, Ndam NT, Ezimegnon S, Alao MJ, Ahouansou C, et al (2014) Cytoadherence phenotype of *Plasmodium falciparum*-infected erythrocytes is associated with specific pfemp-1 expression in parasites from children with cerebral malaria. Malar J 13: 333 10.1186/1475-2875-13-333 25156105 PMC4150962

[20] Almelli T, Nuel G, Bischoff E, Aubouy A, Elati M, et al (2014) Differences in Gene Transcriptomic Pattern of *Plasmodium falciparum* in Children with Cerebral Malaria and Asymptomatic Carriers. PLoS One 9: e114401 10.1371/journal.pone.0114401 25479608 PMC4257676

[21] Merrick CJ, Huttenhower C, Buckee C, Amambua-Ngwa A, Gomez-Escobar N, et al (2012) Epigenetic dysregulation of virulence gene expression in severe *Plasmodium falciparum* malaria. J Infect Dis 205: 1593–1600. 10.1093/infdis/jis239 22448008 PMC3415821

[22] Turner L, Lavstsen T, Berger SS, Wang CW, Petersen JE, et al (2013) Severe malaria is associated with parasite binding to endothelial protein C receptor. Nature 498: 502–505. 10.1038/nature12216 23739325 PMC3870021

[23] Berger SS, Turner L, Wang CW, Petersen JE, Kraft M, et al (2013) *Plasmodium falciparum* expressing domain cassette 5 type PfEMP1 (DC5-PfEMP1) bind PECAM1. PLoS One 8: e69117 10.1371/journal.pone.0069117 23874884 PMC3706608

[24] Sharp S, Lavstsen T, Fivelman QL, Saeed M, McRobert L, et al (2006) Programmed transcription of the *var* gene family, but not of *stevor*, in *Plasmodium falciparum* gametocytes. Eukaryot Cell 5: 1206–1214. 16896206 10.1128/EC.00029-06 PMC1539138

[25] Frank M, Dzikowski R, Amulic B, Deitsch K (2007) Variable switching rates of malaria virulence genes are associated with chromosomal position. Mol Microbiol 64: 1486–1498. 17555435 10.1111/j.1365-2958.2007.05736.x PMC3634120

[26] Falk N, Kaestli M, Qi W, Ott M, Baea K, et al (2009) Analysis of *Plasmodium falciparum var* genes expressed in children from Papua New Guinea. J Infect Dis 200: 347–356. 10.1086/600071 19552523

[27] Enderes C, Kombila D, Dal-Bianco M, Dzikowski R, Kremsner P, et al (2011) *Var* Gene promoter activation in clonal *Plasmodium falciparum* isolates follows a hierarchy and suggests a conserved switching program that is independent of genetic background. J Infect Dis 204: 1620–1631. 10.1093/infdis/jir594 21926380

[28] Gupta S, Snow RW, Donnelly CA, Marsh K, Newbold C (1999) Immunity to non-cerebral severe malaria is acquired after one or two infections. Nat Med 5: 340–343. 10086393 10.1038/6560

[29] Peters J, Fowler E, Gatton M, Chen N, Saul A, et al (2002) High diversity and rapid changeover of expressed *var* genes during the acute phase of *Plasmodium falciparum* infections in human volunteers. Proc Natl Acad Sci U S A 99: 10689–10694. 12142467 10.1073/pnas.162349899 PMC125014

[30] Wang CW, Hermsen CC, Sauerwein RW, Arnot DE, Theander TG, et al (2009) The *Plasmodium falciparum var* gene transcription strategy at the onset of blood stage infection in a human volunteer. Parasitol Int 58: 478–480. 10.1016/j.parint.2009.07.004 19616120

[31] Lavstsen T, Magistrado P, Hermsen CC, Salanti A, Jensen AT, et al (2005) Expression of *Plasmodium falciparum* erythrocyte membrane protein 1 in experimentally infected humans. Malar J 4: 21 15857512 10.1186/1475-2875-4-21 PMC1112614

[32] Bachmann A, Predehl S, May J, Harder S, Burchard GD, et al (2011) Highly co-ordinated *var* gene expression and switching in clinical *Plasmodium falciparum* isolates from non-immune malaria patients. Cell Microbiol 13: 1397–1409. 10.1111/j.1462-5822.2011.01629.x 21740496

[33] Mordmüller B, Supan C, Sim KL, Gómez-Pérez GP, Salazar CLO, et al (2015) Direct venous inoculation of *Plasmodium falciparum* sporzoites for controlled human malaria infection: a dose-finding trial in two centres. Malar J 14. 10.1186/s12936-015-0628-0 PMC4371633 25889522

[34] Sauerwein RW, Roestenberg M, Moorthy VS (2011) Experimental human challenge infections can accelerate clinical malaria vaccine development. Nat Rev Immunol 11: 57–64. 10.1038/nri2902 21179119

[35] Fastman Y, Noble R, Recker M, Dzikowski R (2012) Erasing the epigenetic memory and beginning to switch-the onset of antigenic switching of *var* genes in *Plasmodium falciparum* . PLoS One 7: e34168 10.1371/journal.pone.0034168 22461905 PMC3312910

[36] Dzikowski R, Frank M, Deitsch K (2006) Mutually exclusive expression of virulence genes by malaria parasites is regulated independently of antigen production. PLoS Pathog 2: e22 16518466 10.1371/journal.ppat.0020022 PMC1386720

[37] Warimwe GM, Recker M, Kiragu EW, Buckee CO, Wambua J, et al (2013) *Plasmodium falciparum var* gene expression homogeneity as a marker of the host-parasite relationship under different levels of naturally acquired immunity to malaria. PLoS One 8: e70467 10.1371/journal.pone.0070467 23922996 PMC3726600

[38] Horrocks P, Pinches R, Christodoulou Z, Kyes SA, Newbold CI (2004) Variable *var* transition rates underlie antigenic variation in malaria. Proc Natl Acad Sci U S A 101: 11129–11134. 15256597 10.1073/pnas.0402347101 PMC503751

[39] Blomqvist K, Normark J, Nilsson D, Ribacke U, Orikiriza J, et al (2010) *var* gene transcription dynamics in *Plasmodium falciparum* patient isolates. Mol Biochem Parasitol 170: 74–83. 10.1016/j.molbiopara.2009.12.002 20006652

[40] Amulic B, Salanti A, Lavstsen T, Nielsen MA, Deitsch KW (2009) An upstream open reading frame controls translation of *var2csa*, a gene implicated in placental malaria. PLoS Pathog 5: e1000256 10.1371/journal.ppat.1000256 19119419 PMC2603286

[41] Wang CW, Mwakalinga SB, Sutherland CJ, Schwank S, Sharp S, et al (2010) Identification of a major *rif* transcript common to gametocytes and sporozoites of *Plasmodium falciparum* . Malar J 9: 147 10.1186/1475-2875-9-147 20509952 PMC2890677

[42] Spence PJ, Jarra W, Levy P, Reid AJ, Chappell L, et al (2013) Vector transmission regulates immune control of *Plasmodium* virulence. Nature 498: 228–231. 10.1038/nature12231 23719378 PMC3784817

[43] Frech C, Chen N (2013) Variant surface antigens of malaria parasites: functional and evolutionary insights from comparative gene family classification and analysis. BMC Genomics 14: 427 10.1186/1471-2164-14-427 23805789 PMC3747859

[44] Brannan LR, McLean SA, Phillips RS (1993) Antigenic variants of *Plasmodium chabaudi chabaudi AS* and the effects of mosquito transmission. Parasite Immunol 15: 135–141. 8100357 10.1111/j.1365-3024.1993.tb00593.x

[45] McLean SA, Phillips RS, Pearson CD, Walliker D (1987) The effect of mosquito transmission of antigenic variants of *Plasmodium chabaudi* . Parasitology 94 (Pt 3): 443–449. 3614987 10.1017/s0031182000055797

[46] Trager W, Jensen JB (1976) Human malaria parasites in continuous culture. Science 193: 673–675. 781840 10.1126/science.781840

[47] Lambros C, Vanderberg JP (1979) Synchronization of *Plasmodium falciparum* erythrocytic stages in culture. J Parasitol 65: 418–420. 383936

[48] Duffy MF, Byrne TJ, Carret C, Ivens A, Brown GV (2009) Ectopic recombination of a malaria *var* gene during mitosis associated with an altered *var* switch rate. J Mol Biol 389: 453–469. 10.1016/j.jmb.2009.04.032 19389407 PMC3898907

