# Supplementary material for: Mosquito Passage Dramatically Changes var Gene Expression in Controlled Human Plasmodium falciparum Infections
Source: PLoS Pathog. 2016 Apr 12;12(4):e1005538. doi: 10.1371/journal.ppat.1005538 (PMC4829248; doi:10.1371/journal.ppat.1005538)
Supplement: S1 Table — (PDF) [file ppat.1005538.s001.pdf]

Table S1: Primer sets used for qPCR analysis to amplify the entire NF54 *var* gene repertoire

Primer ID	New gene ID	Gene ID	Forward primer (5'→3')	Reverse primer (5'→3')	E
AS1 [1]	PF3D7_0100100	PFA0005w	TGCGCTGATAACTCACAACA	AGGGGTTCATCGTCATCTTC	2.07
AS9 [1]	PF3D7_1200400	PFL0020w	TCGATTATGTGCCGCAGTAT	TTCCCGTACAATCGTATCCA	2.03
AS13 [1]	PF3D7_1240400, PF3D7_1240900	PFL1955w, PFL1970w	AAAGCCACTAGCGAGGGTAA	TGTTTTTGCCCACTCCTGTA	2.03
AS21 [1]	PF3D7_0223500	PFB1055c	CAATTTTGGGTGTGGAATCA	CACTGGCCACCAAGTGTATC	2.04
AS27 [1]	PF3D7_0412700	PFD0625c	TAAAAGACGCCAACAGATGC	TCATCGTCTTCGTCTTCGTC	2.10
AS34 [1]	PF3D7_0421300	PFD1015c	TGCAACGAAACATTAGCACA	AGCAGGGGATGATGCTTTAC	1.97
AS39 [1]	PF3D7_0617400	MAL6P1.252	ATTTGTCGCACATGAAGGAA	AACTTCGTGCCAATGCTGTA	1.99
AS46 [1]	PF3D7_0712400	PF07_0050	GCGACGCTCAAAAACATTTA	TCATCCAACGCAATCTTTGT	2.02
AS10 [1]	PF3D7_1200600	PFL0030c	TGGTGATGGTACTGCTGGAT	TTTATTTTCGGCAGCATTTG	2.02
AS15 [1]	PF3D7_1240600	PFL1960w	CATCCATTACGCAGGATACG	AAATAGGGTGGGCGTAACAC	2.06
AS22 [1]	PF3D7_0200100	PFB0010w	ATGTGCGCTACAAGAAGCTG	TTGATCTCCCCATTCAGTCA	1.97
AS28 [1]	PF3D7_0412900, PF3D7_0413100	PFD0630c, PFD0635c	ACTTTCTGGTGGGGAATCAG	TTCACCGCCACTTACTTCAG	2.10
AS36 [1]	PF3D7_0426000	PFD1245c	TGACGACTCCTCAGACGAAG	CTCCACTGACGGATCTGTTG	2.01
AS43 [1]	PF3D7_0800100	PF08_0142	GTCGTGGAAAAACGAAAGGT	TATCTATCCAGGGCCCAAAG	1.97
AS5 [1]	PF3D7_1041300	PF10_0406	GTGCACCAAAAGAAGCTCAA	ACAAAACTCCTCTGCCCATT	2.00
AS11 [1]	PF3D7_1219300	PFL0935c	GACGCCTGCACTCTCAAATA	TTGGAGAGCACCACCATTTA	2.07
AS18 [1]	PF3D7_1200100	PFL0005w	CGGAGGAGGAAAAACAAGAG	TGCCGTATTTGAGACCACAT	2.06
AS23 [1]	PF3D7_0324900, PF3D7_0300100	PFC1120c, PFC0005w	CAATCTGCGGCAATAGAGAC	CCACTGTTGAGGGGTTTTCT	2.03
AS30 [1]	PF3D7_0400100	PFD0005w	GACGACGATGAAGACGAAGA	AGATCTCCGCATTTCCAATC	2.07
AS37* [1]	PF3D7_0533100	PFE1640w	AAGAAAGTGCCACAACATGC	GTTCGTACGCCTGTCGTTTA	2.05
AS44 [1]	PF3D7_0632500	MAL6P1.4	ATGTGTGCGAGAAGGTGAAG	TGCCTTCTAGGTGGCATACA	2.03
AS50 [1]	PF3D7_0712900	MAL7P1.56	CACACATGTCCACCACAAGA	ACCCTTCTGTGGTGTCTTCC	2.02
AS6 [1]	PF3D7_1100100	PF11_0007	GAGGCTTATGGGAAACCAGA	AGGCAGTCTTTGGCATCTTT	2.07
AS19 [1]	PF3D7_1373500	PF13_0364	CGGAATTAGTTGCCTTCACA	CATTGGCCACCAAGTGTATC	2.00
AS26 [1]	PF3D7_0412400	PFD0615c	ACCGCCCCATCTAGTGATAG	CACTTGGTGATGTGGTGTCA	2.02
AS31 [1]	PF3D7_0420700, PF3D7_0420900	PFD0995c, PFD1000c	AGAGGGTTATGGGAATGCAG	GCATTCTTTGGCAATTCCTT	2.06
AS45 [1]	PF3D7_0711700	PF07_0048	CAATTTTTCCGACGCTTGTA	CACATATAGCGCCGTCCTTA	2.06
AS51 [1]	PF3D7_0712800	MAL7P1.55	ACGTGGTGGAGACGTAAACA	CCTTTGTTGTTGCCACTTTG	2.01
AS91 [1]	PF3D7_1300100	PF13_0001	ACAAAGGAACGTCCATCTCC	GCCAATACTCCACATGATCG	1.99
AS95 [1]	PF3D7_0420700	PFD0995c	TCACAACCTGACCCCCTACT	TCTTCGTCGTTGTCATCCTC	2.01
AS53 [1]	PF3D7_0733000	PF07_0139	TGACGACGATAAATGGGAAA	TTCTTTTGGAGCAGGGAGTT	1.91
AS92 [1]	PF3D7_0809100	PF08_0103	TGCAAGGGTGCTAATGGTAA	CCTGCATTTTGACATTCGTC	1.98
AS60b** [1]	PF3D7_0717700	PF07_0073	AAGTAGCAGGTCATCGTGGTT	TTCGGCACATTCTTCCATAA	1.96
AS55 [1]	PF3D7_0808700	PF08_0106	TTTGTCCGGAAGACGATACA	ATCTGGGGCAGAATTACCAC	1.99
AS93 [1]	PF3D7_0632800	MAL6P1.1	GACAAATACGGCGACTACGA	TGTTTCACCCCATTCTTCAA	1.97
AS61b** [1]	PF3D7_1444800	PF14_0425	TGTACCACCAGCCTTACCAG	TTCCTTGCCATGTGTTCAAT	1.92
AS56 [1]	PF3D7_0900100	PFI0005w	TGCAAACCACCAGAAGAAAG	GTTCTCCGTGTTGTCCTCCT	2.01
AS7 [1]	PF3D7_1100200	PF11_0008	GACGGCTACCACAGAGACAA	CGTCATCATCGTCTTCGTTT	1.96
AS8 [1]	PF3D7_1150400	PF11_0521	TGCTGAAGACCAAATTGAGC	TTGTTGTGGTGGTTGTTGTG	1.95
AS20 [1]	PF3D7_1300300	PF13_0003	CACAGGTATGGGAAGCAATG	CCATACAGCCGTGACTGTTC	1.91
AS25 [1]	PF3D7_0400400	PFD0020c	ATATGGGAAGGGATGCTCTG	TGAACCATCGAAGGAATTGA	1.94
AS35 [1]	PF3D7_0425800	PFD1235w	AAACACGTTGAATGGCGATA	GACGCCGAGGAGGTAAATAG	1.90
AS41 [1]	PF3D7_0800200	PF08_0141	GGTGTCAAGGCAGCTAATGA	TATGTCCTGCGCTATTTTGC	1.92
AS96 [1]	PF3D7_0937600	PFI1820w	TGACCAAGACGAAGTATGGAA	TTGATCTCTGTTCGCTGTCC	1.95
AS97 [1]	PF3D7_0100300	PFA0015c	TCATTATGGGAAGCACGATT	TGATTTCTACCATCGCAAGG	1.96
AS4 [1]	PF3D7_0421104	PF10_0001	GACGAGGAGTCGGAAAAGAC	TGGACAGGCTTGTTTGAGAG	1.96
WT1 [2]	PF3D7_0600400	PFF0020c	GCACATTATCAAACGCCC	AACCAGCTGCCTTGTGCAA	2.00
RD1 [3]	PF3D7_0808600	PF08_0107	CCTAAAAAGGACGCAGAAGG	CCAGCAACACTACCACCAGT	1.94
RD4** [3]	PF3D7_1218600	PFL0900c	AAGAGATGCATGTTGGTC	GTACCCCAATCACCTACA	1.97
TL1 [4]	PF3D7_0937800	PFI1830c	ACAACAATTTCGCAAGCAAG	TTCCTCTGCCTCCTCTTCAT	1.90
MF2 [5]	PF3D7_1255200	PFL2665c	GCGAGGTCTTCTCGTTCTTG	ATGACGAAGAAGCAGCAGGT	2.00
MF3 [5]	PF3D7_0800300	PF08_0140	GGAGGAGGAAGAGGAAAACG	CCACCTCCTCTTGTTGTGGT	1.97
MF4 [5]	PF3D7_0712300	MAL7P1.50	GGTGGAGGTAGTCCACAGGA	CAGCTATTTCCCCACCAGAA	1.93
MDa101 [6]	PF3D7_0115700	PFA0765c	CAAGAGACACAACCGGAAGA	CACTTCCAATTGGGGAATTT	2.03
MDa500/501 [6]	PF3D7_0700100	MAL8P1.220	GTCCTCTATGTGGAGTGAAAA AGAA	AGTACCGTTATCTGGGTTTAT AGGC	2.07
MDa498/499 [6]	PF3D7_0833500	MAL7P1.212	AATCAGAAAAGTGTAATTGCA GGAG	TTTACTATCATCACTGACACG CATT	1.97
MD275/276 [7]	PF3D7_0600200	MAL6P1.316	TGATGGTACTTGTATTGAAGC	TGGAGTAGGCGTCTCATCTCG	2.00

			ACAGA		
MD93/94*** [7]	PF3D7_0501300	PFE0065w	TTAGCCGACGAACCAACACA	TTCGGTTGTCTCTGGTACTGCA	2.01
MD273/274 [7]	PF3D7_0421100	PFD1005c	TGGCGTCCACTCCGACA	GAGCCTTCGGATTCATCGTG	1.98
AB1	PF3D7_0712000	PF07_0049	ATGAATTTGGGCAAAAAGTGT ACG	TCATTCCAAATTGGTGCTAGTGA	2.00
AB2	PF3D7_1240300	PFL1950w	ACGCAGAAGTACAAAGAGATGC	ATCCGGTGATGTCGTTCCTT	2.12
AB3	PF3D7_0500100	PFE0005w	GAGTGGTGGTAACACGGAGA	ATCTTGTGACGCAGTTTGGG	2.07
AB4	PF3D7_0712600	PF07_0051	TGCACGACCAAATGAAAAAGGA	ATCGGTGGCACCTGTTTCTC	1.95

E, amplification efficiency
* pseudogene
** housekeeping genes
*** sbp1 (normalizer)

References

1. Salanti A, Staalsoe T, Lavstsen T, Jensen AT, Sowa MP, et al. (2003) Selective upregulation of a single distinctly structured *var* gene in chondroitin sulphate A-adhering *Plasmodium falciparum* involved in pregnancy-associated malaria. Mol Microbiol 49: 179-191.
2. Tham WH, Payne PD, Brown GV, Rogerson SJ (2007) Identification of basic transcriptional elements required for *rif* gene transcription. Int J Parasitol 37: 605-615.
3. Dzikowski R, Frank M, Deitsch K (2006) Mutually exclusive expression of virulence genes by malaria parasites is regulated independently of antigen production. PLoS Pathog 2: e22.
4. Lavstsen T, Magistrado P, Hermsen CC, Salanti A, Jensen AT, et al. (2005) Expression of *Plasmodium falciparum* erythrocyte membrane protein 1 in experimentally infected humans. Malar J 4: 21.
5. Frank M, Dzikowski R, Amulic B, Deitsch K (2007) Variable switching rates of malaria virulence genes are associated with chromosomal position. Mol Microbiol 64: 1486-1498.
6. Dahlback M, Lavstsen T, Salanti A, Hviid L, Arnot DE, et al. (2007) Changes in *var* gene mRNA levels during erythrocytic development in two phenotypically distinct *Plasmodium falciparum* parasites. Malar J 6: 78.
7. Petter M, Lee CC, Byrne TJ, Boysen KE, Volz J, et al. (2011) Expression of *P. falciparum var* genes involves exchange of the histone variant H2A.Z at the promoter. PLoS Pathog 7: e1001292.
