# Supplementary figures and images for: Mosquito Passage Dramatically Changes var Gene Expression in Controlled Human Plasmodium falciparum Infections
Source: PLoS Pathog. 2016 Apr 12;12(4):e1005538. doi: 10.1371/journal.ppat.1005538 (PMC4829248; doi:10.1371/journal.ppat.1005538)

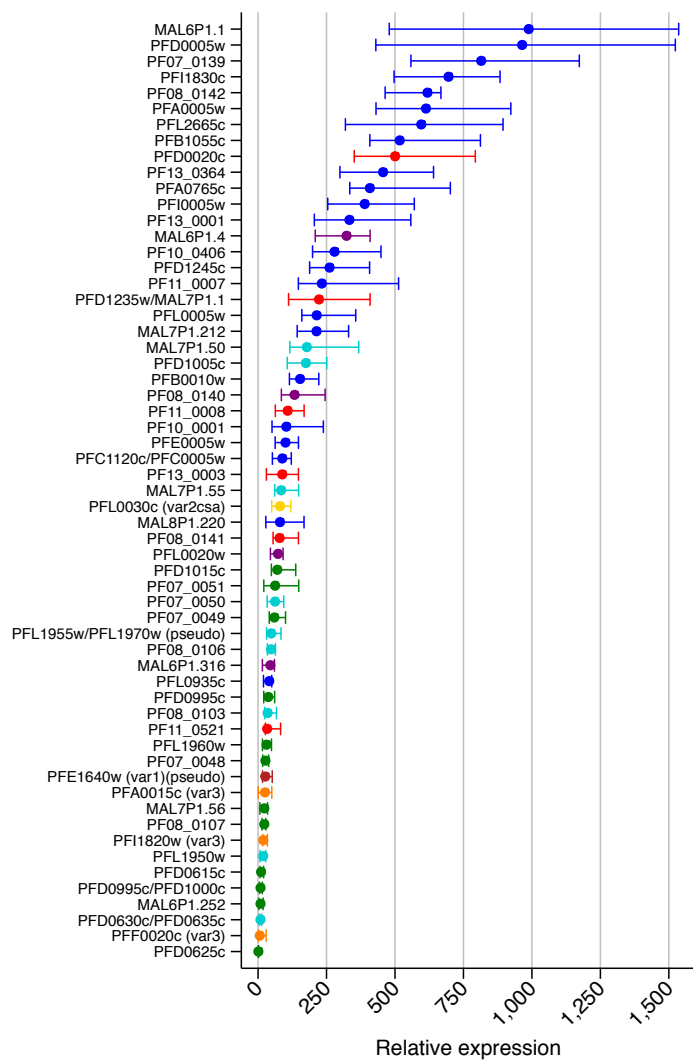

Supplement: S2 Fig — The median var transcript level relative to the sbp1 transcript level with IQR is shown for all 18 volunteer samples. Expression values for genes of the different var groups are presented in red (A), orange (subfamily var3), dark red (subfamily var1), purple (B/A), blue (B), turquoise (B/C), green (C) and yellow (E). (PDF) [file ppat.1005538.s003.pdf]

**A**

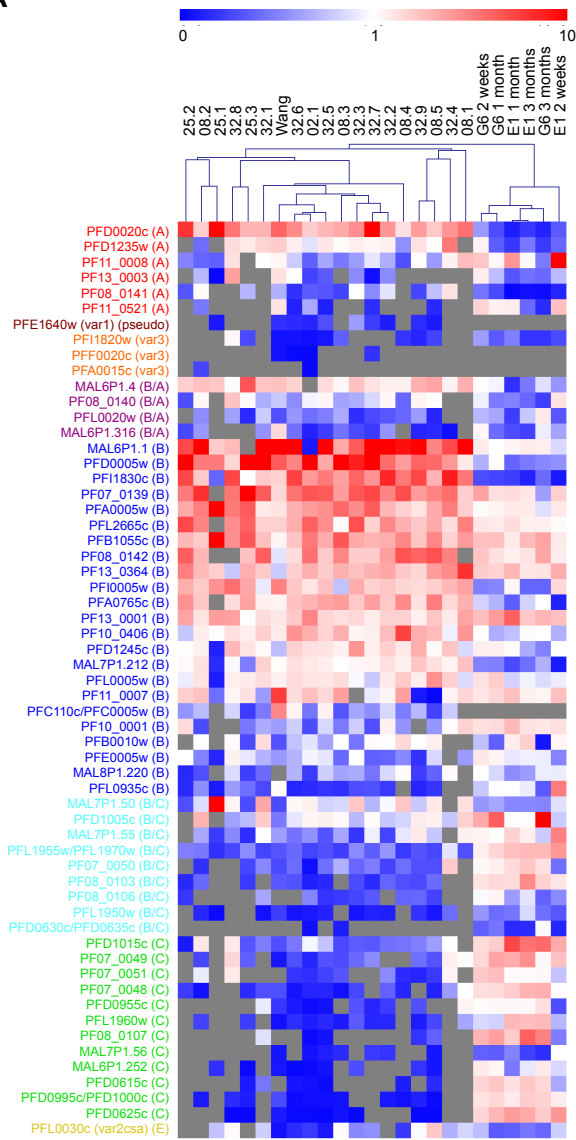

**B**

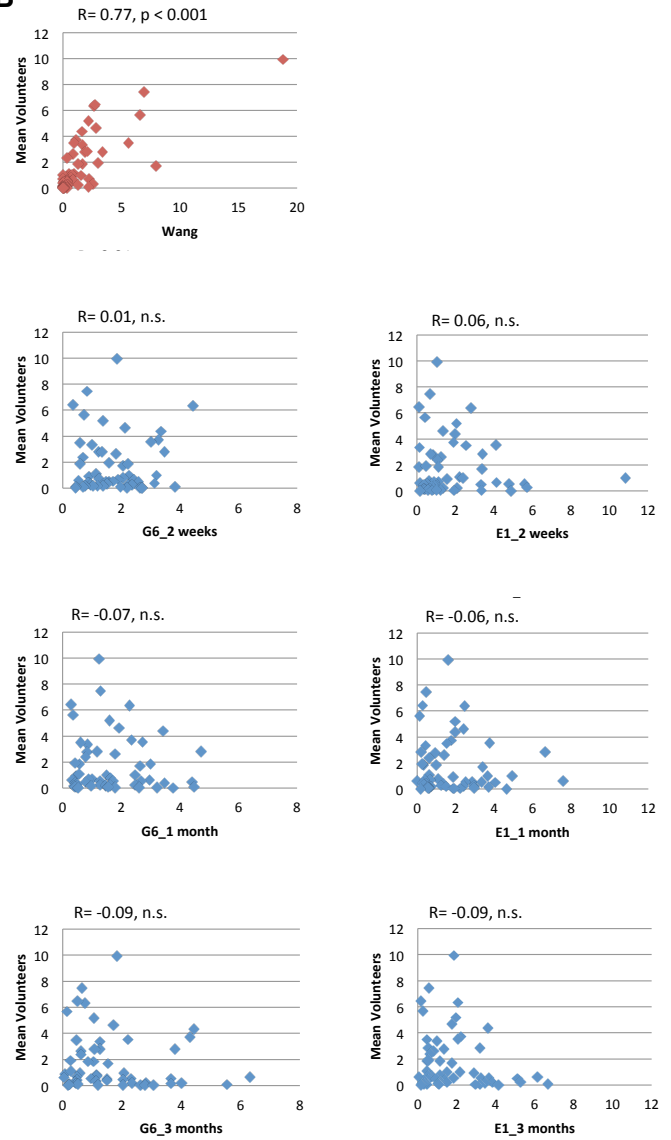

**C**

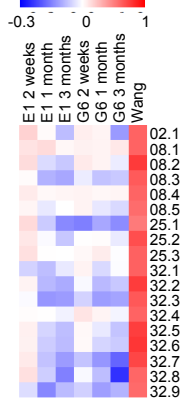

Supplement: S3 Fig — (A) Heat map showing the individual var gene expression profiles for all volunteer samples on the day of patent infection and for the volunteer sample obtained by Wang et al. [30]. Furthermore, var gene expression data is shown for two 3D7 cell lines (E1 and G6), in which a “null-var” phenotype was created in vitro, var gene expression was reactivated and analyzed two weeks, one and three months after drug removal [35]. Var genes are ranked by var gene group and mean expression. To correct for individual differences in the overall var expression levels and for the use of different normalizing genes in the three studies, the expression of each var gene was normalized against the total var expression in each sample. Samples were hierarchically clustered using average linkage clustering with Spearman’s Rank Correlation as distance metric. The color scale indicates the relative expression levels with red representing values above the median, blue representing values below the median, and white representing median. Grey means not detected. (B) Scatter plots of normalized var gene expression data obtained by Wang et al. [30] and Fastman et al. [35] against mean var gene expression from all ex vivo volunteer samples at the day of first microscopically detectable parasitemia. Spearman’s rank correlation coefficient (R) and significances (p-values) are indicated above each graph. (C) Heat map of pairwise Spearman’s rank correlation coefficients (R) of var gene expression profiles from Wang et al. and Fastman et al. [30,35] against the mean ex vivo var gene expression of all volunteers at the day of patent infection. The color scale indicates the correlation coefficient with red indicating positive correlation, blue indicating negative correlation and white indicating no correlation. (PDF) [file ppat.1005538.s004.pdf]
