# Supplementary material for: Mosquito Passage Dramatically Changes var Gene Expression in Controlled Human Plasmodium falciparum Infections
Source: PLoS Pathog. 2016 Apr 12;12(4):e1005538. doi: 10.1371/journal.ppat.1005538 (PMC4829248; doi:10.1371/journal.ppat.1005538)
Supplement: S1 Fig — Spearman’s rank correlation coefficient (R) and significances (p-values) are indicated above each graph. (PDF) [file ppat.1005538.s002.pdf]

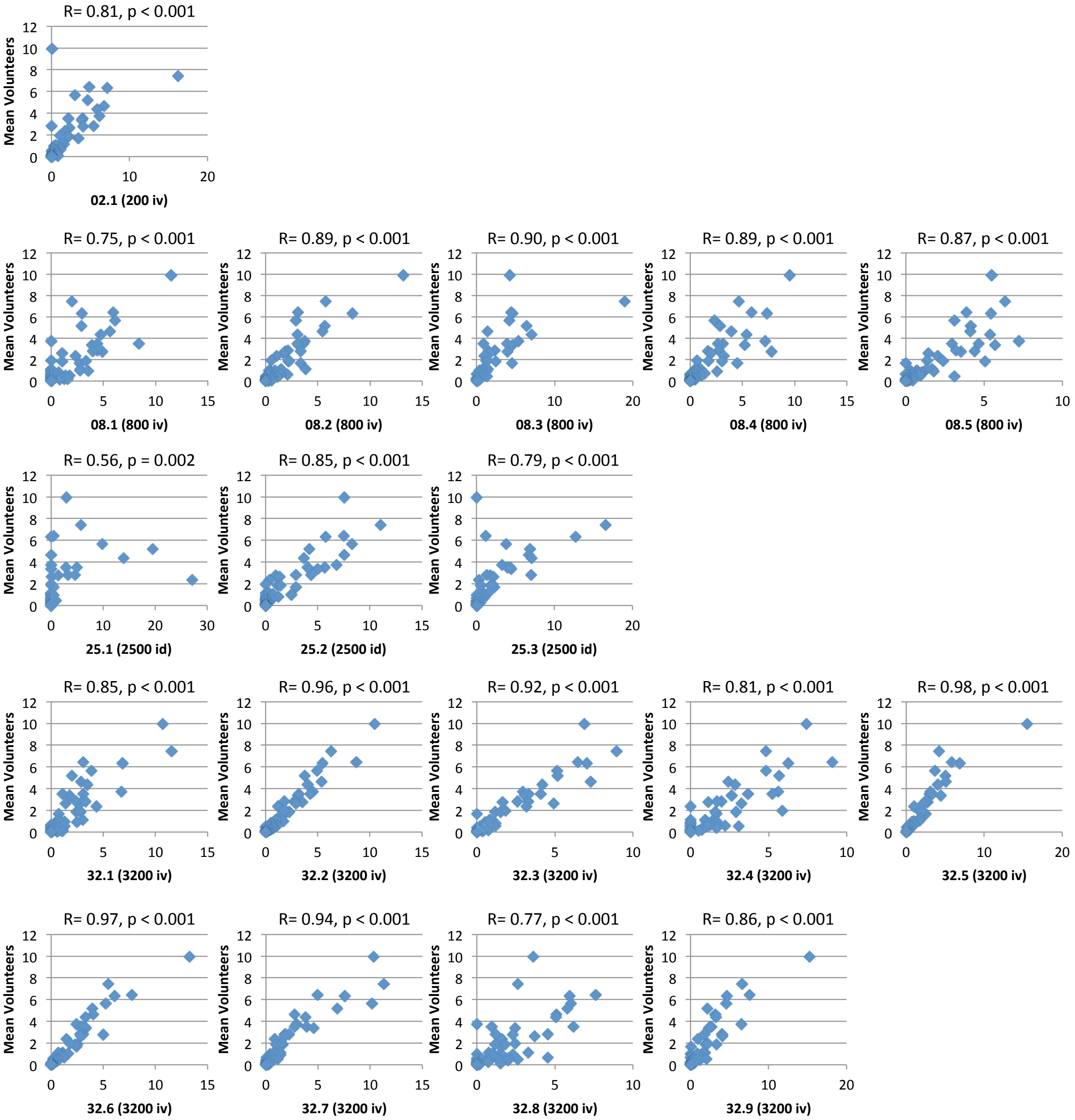
R= 0.81, p < 0.001
Mean Volunteers
02.1 (200 iv)
R= 0.75, p < 0.001
08.1 (800 iv)
R= 0.89, p < 0.001
08.2 (800 iv)
R= 0.90, p < 0.001
08.3 (800 iv)
R= 0.89, p < 0.001
08.4 (800 iv)
R= 0.87, p < 0.001
08.5 (800 iv)
R= 0.56, p = 0.002
25.1 (2500 id)
R= 0.85, p < 0.001
25.2 (2500 id)
R= 0.79, p < 0.001
25.3 (2500 id)
R= 0.85, p < 0.001
32.1 (3200 iv)
R= 0.96, p < 0.001
32.2 (3200 iv)
R= 0.92, p < 0.001
32.3 (3200 iv)
R= 0.81, p < 0.001
32.4 (3200 iv)
R= 0.98, p < 0.001
32.5 (3200 iv)
R= 0.97, p < 0.001
32.6 (3200 iv)
R= 0.94, p < 0.001
32.7 (3200 iv)
R= 0.77, p < 0.001
32.8 (3200 iv)
R= 0.86, p < 0.001
32.9 (3200 iv)
