# Supplementary material for: Mosquito Passage Dramatically Changes var Gene Expression in Controlled Human Plasmodium falciparum Infections
Source: PLoS Pathog. 2016 Apr 12;12(4):e1005538. doi: 10.1371/journal.ppat.1005538 (PMC4829248; doi:10.1371/journal.ppat.1005538)
Supplement: S4 Fig — (A) var gene expression in parasites recovered from volunteer 02.1, who received 200 sporozoites intravenously 14 days before. Shown are transcript levels of all NF54 var genes and three housekeeping controls as indicated. For comparison, the median expression value calculated from all volunteer samples is displayed for each gene as a black line. Both genes with remarkably low expression values, MAL6P1.4/PF3D7_0632500 and MAL6P1.1/PF3D7_0632800, are highlighted in red boxes. (B) var qPCR analysis with genomic DNA extracted from two vials of Master Cell Bank parasites (MCB-A and -B), Working Cell Bank parasites (WCB) and parasites recovered from volunteers 02.1 (VOL 02.1) and 32.5 (VOL 32.5). Shown are Ct (cycle of treshold) values for each individual var gene primer pair. The primer pair for the variant MAL6P1.4 failed to detect an amplification product in the sample from volunteer 02.1 within 40 PCR cycles (Ct ≥ 40). An increased Ct value for the variant MAL6P1.1 of 5.3 PCR cycles reflects a reduction of 38.9 fold on genomic DNA level (2ΔCt). (PDF) [file ppat.1005538.s005.pdf]

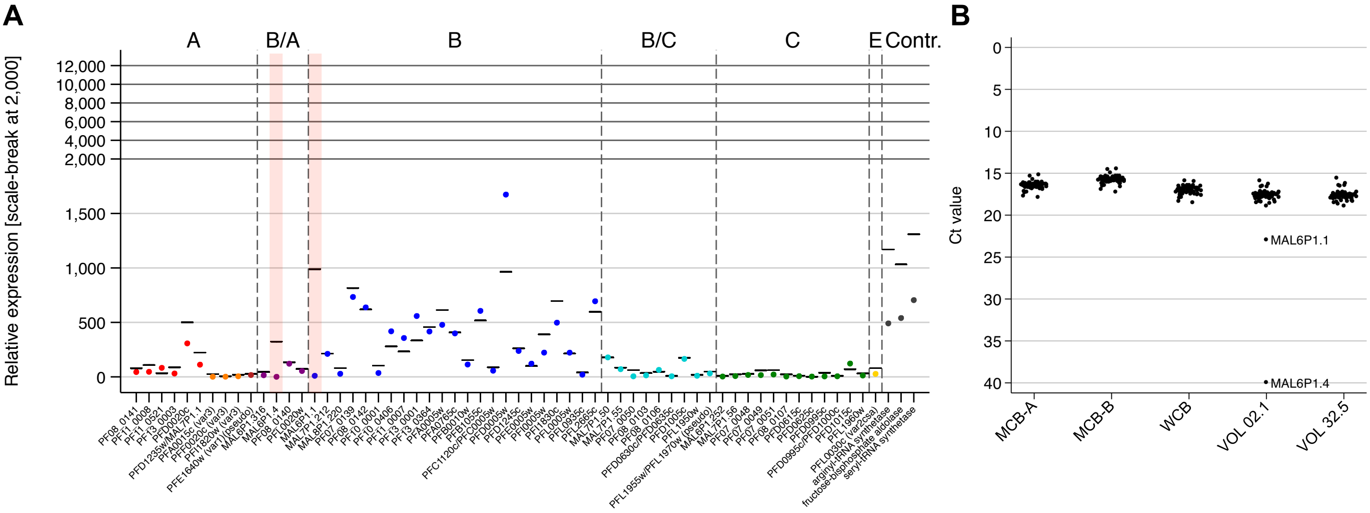
A
B
A
B/A
B
B/C
C
E
Contr.
Relative expression [scale-break at 2,000]
12,000
10,000
8,000
6,000
4,000
2,000
1,500
1,000
500
0
Ct value
0
5
10
15
20
25
30
35
40
MAL6P1.1
MAL6P1.4
MCB-A
MCB-B
WCB
VOL 02.1
VOL 32.5
